# Trends in reproductive and maternal healthcare indicators, Côte d’Ivoire, 2018 to 2020

**DOI:** 10.4102/jphia.v16i1.1301

**Published:** 2025-07-17

**Authors:** Denise O.D. Kpebo, Marie-Dorothée Mélèdje Koffi-Koumi, Marie-Laurette Agbré-Yacé, Jean-Ernest D. Kamenan, Odile S. Aké-Tano, William Yavo, Gouda R.M. Mady, Diarra B. Senghor, Cheikh M. Faye

**Affiliations:** 1 Maternal and Child Health Unit, National Public Health Institute, Abidjan, Côte d’Ivoire; 2 Department of Public Health, Medical School, University Allassane Ouattara, Abidjan, Côte d’Ivoire; 3 Department of Maternal and Reproductive Health, Unit of Research in Reproductive Health of Côte d’Ivoire, Abidjan, Côte d’Ivoire; 4 Department of Health Information, Service of Health Management Information and Statistics, Abidjan, Côte d’Ivoire; 5 Department of Management and Administration, National Public Health Institute, Abidjan, Côte d’Ivoire; 6 Project Monitoring and Evaluation, African Population Health Research Center, Dakar, Senegal; 7 Management and Administration Unit, African Population Health Research Center, Dakar, Senegal

**Keywords:** maternal health, reproductive health, modern contraceptive, trends, Côte d’Ivoire

## Abstract

**Background:**

Despite some improvement, there are still persistent challenges in the area of reproductive and maternal health in Côte d’Ivoire.

**Aim:**

Analysing subnational trends of key indicators of reproductive and maternal healthcare from 2018 to 2020, regarding the targets of the investment case of 2023.

**Setting:**

All 33 health regions and 113 health districts (HDs).

**Methods:**

The analysis was concerned with four indicators: the modern contraceptive prevalence rate (mCPR), the proportion of first antenatal care (ANC1) during the first trimester, the coverage of four antenatal care (ANC4), and the coverage of delivery with a skilled birth attendant (SBA). Using data from the national health information system, we analysed the evolutive trends of each indicator at district level, assessed the performance regarding the 2023 targets, and identified the disparities at districts and regions level. Data were processed using Microsoft Excel, QGIS 3.16 and Stata 15.0 software.

**Results:**

There was a lack of significant progress for mCPR and ANC1 during the first trimester. In 2020, more than two out of three of the HDs were still far from the 2023 target for both indicators. In contrast, there was a clear progression for ANC4 and SBA, with more than 40% of districts having already reached the 2023 target. These evolutive trends were associated with high disparities across HDs and regions.

**Conclusion:**

In spite of positive evolutive trends for some indicators, there are still high disparities at subnational levels.

**Contribution:**

Interventions need to be fully implemented, and progress monitoring should be reinforced and pursued for timely adjustments.

## Introduction

Globally, there is renewed support for reproductive, maternal, newborn, child and adolescent health (RMNCAH) as part of the Sustainable Development Goals (SDGs) and the Global Strategy for Women’s, Children’s, and Adolescent’s Health.^1,2^ The Global Strategy aims to achieve the highest attainable standard of health for all women, children and adolescents and to ensure that every newborn, mother and child not only survives but thrives.^2^

In the specific context of Côte d’Ivoire, the last decades have been marked by a notable improvement in the health and survival of the mother-child couple.^3^ However, despite this notable progress, the country still faces persistent challenges in the area of reproductive, maternal, neonatal, child and adolescent health and nutrition (RMNCAH+N). The results of a recent literature review of RMNCAH+N programme indicators along with those of a recent qualitative study support these facts.^4,5^ The Government of Côte d’Ivoire, with a view to remedying these shortcomings and guaranteeing equitable access to quality care for all, has launched, with the support of partners, several reforms and initiatives to improve access to quality maternal, neonatal and child services.^6^

In order to strengthen such national initiatives, international commitments such as the Countdown to 2030 (CD 2030) initiative provide a vehicle to enhance both domestic and external support for RMNCAH in sub-Saharan African countries.^7,8^ The objective is to ensure smart, scaled-up and sustained financing with a focus on achieving targeted results. The CD 2030 initiative has been monitoring progress since 2005 for life-saving interventions in RMNCAH+N.^8,9^ The last phase of CD 2030 (2020–2022) was focused on collaboration and technical support for approximately 15–20 countries supported by the Global Financing Facility (GFF), including Côte d’Ivoire. The objective of this phase was to monitor the performance of RMNCAH+N indicators in Côte d’Ivoire, with a focus on the national investment case.^10,11^

The implementation of the investment case started in 2020 and was planned to continue until 2024. It consisted of the implementation of interventions relating to the strengthening of community activities, the improvement of the supply of care, the strengthening of the national health information system and the supply chain.^6,10,11^

With the aim of monitoring the impact of all these interventions on the performance of key RMNCAH+N indicators, the CD 2030 project also started in September 2020 in Côte d’Ivoire. Then, as part of CD 2030, this work was carried out with the aim of analysing subnational progress and performance (health districts [HDs] and regions) of four key indicators of reproductive and maternal healthcare from 2018 to 2020 in Côte d’Ivoire. These are the modern contraceptive prevalence rate (mCPR), the proportion of ANC1 in the first trimester (ANC1-T1), the coverage of ANC4, and the coverage of births attended by skilled health attendant (SBA). These four key indicators, relevant for maternal healthcare, are part of the SDG 3, which aims at ensuring healthy lives and promoting well-being for all at all ages. For instance, the SDG 3.1.2 is specifically concerned with SBA, while the SDG 3.7.1 is concerned with mCPR. Finally, the coverage of ANC is included in the SDG 3.8.1.^2^

More specifically, this analysis was intended to (1) describe the spatial distribution of the priority indicators of maternal healthcare and (2) describe the spatio-temporal evolutive trends of the priority indicators at the level of the regions and HDs.

The results should help guide policymakers in adjusting the interventions implemented under the national 2020–2023 investment case to improve mother and child health in Côte d’Ivoire.

## Research methods and design

### Type of study and data sources

This was a quantitative analysis of secondary data. The main source of data to assess the RMNCAH+N indicators progress was the national Health Management Information System (HMIS). For all 113 HDs, monthly routine data related to maternal healthcare for the years 2018, 2019 and 2020 were obtained from the HMIS on 27 April 2022. An Microsoft Excel^®^ data extraction template was developed and shared with the team in charge of extracting the data. It specified the period and the list of the four indicators concerned by this analysis as detailed below:

- 01 indicator of reproductive healthcare, namely the coverage of the mCPR.
- 03 indicators of maternal healthcare, namely ANC-T1, the coverage of 4 antenatal care (ANC4) visits, and the coverage of births attended by an SBA.

### Data quality assessment

Data quality assessments were performed for the routine data using a set of methods previously developed by the World Health Organization (WHO) and the CD 2030,^10^ using Stata 15.0 software. The level of reporting completeness, extreme outliers and internal consistency over time and denominator populations were assessed. Data to be analysed were adjusted based on these data issues.

#### Evaluation and corrections of extreme outliers

In general, limited year-to-year variation in the number of interventions is expected, especially for high-coverage interventions (e.g., ANC1, Penta1). In the presence of large annual variations without a plausible explanation, the data are considered ‘noisy’ and can present serious data quality issues. The extreme values or outliers that could affect the indicator coverage were assessed using a modified *Z*-score (a standardised score of observations measuring the deviation from the median) that was obtained by dividing the difference from the median by median absolute deviation. Monthly data with a score higher than five standard deviations from the annual median were identified as outliers. Extreme outliers were corrected by inputting a value based on the median of 6 months before and 6 months after the outlying values.

#### Evaluation of internal consistency

Internal consistency was assessed using the ANC1 and the first dose of the pentavalent vaccination by comparing the trend of reported numbers with that of expected numbers based on a log-transformed regression analysis (*ln*[*y*] = *ax* + *b*, where *y* is the reported number and *x* is the year). In the case of the near-universal coverage interventions, the increases are only driven by population growth. We expected the slope of the regression to be about 3% (within the range of 1% – 4.9%) and the year-to-year fluctuations, as measured by the standard errors of the regression line, to be small if data quality was good.

#### Missing values for the volume of services

Missing values were inputted using the median value of 6 months before and 6 months after, unless there was reason to believe that it was a true missing value because service was not provided.

#### Summary of data quality assessment

Completeness of reporting was at least 90% over the years for all indicators, but all facilities had missing monthly data and extreme outliers. In Côte d’Ivoire, this situation could be explained by the redistribution recently carried out at the level of the HD. Indeed, from 2017 to 2019, the country had 86 districts spread over 20 health regions. Then from 2020, it went to 113 districts and 33 health regions. As a result, the 27 new districts, although taken into account in this analysis, did not in fact carry out any activities from 2017 to 2019.

### Completeness of reporting

For this analysis, the reporting rate was considered acceptable when it was at least equal to 90%. A summary of the percentage of HDs with monthly reporting rates below 90% was produced alongside a list of all districts with reporting rates below 75%. In all instances where the reporting rate was below 75%, the value of the month would be replaced by the median value of the year. The analysis adjusted for non-reporting facilities by using the adjustment factor *k*. This factor takes into account the level of health services expected among facilities that did not report data (including those in the private sector). An adjustment factor was assigned for each health service based on the knowledge of the programme and by assuming that non-reporting facilities provided certain services as follows:

- *k* = 0.75 for first antenatal care (ANC1): Almost as much service provided in non-reporting health facilities as in reporting health facilities.
- *k* = 0.75 for deliveries: Almost as much service provided in non-reporting health facilities as in reporting health facilities.
- *k* = 0.1 for family planning (FP): About 10% of the amount of health service provided in reporting health facilities is provided by non-reporting health facilities.

### Data management and analysis

#### Mapping

According to the available database, 3 maps were produced for each indicator, which gives a total of 12 maps according to the HD distribution. Regarding the legend of the maps, three intervals have been developed for each indicator according to the targets set at the national level to be achieved in 2023 and 2030:

- Interval 1: < target 2023.
- Interval 2: target 2023 – target 2030.
- Interval 3: ≥ target 2030.

The colour assigned to the intervals gradually becomes lighter to illustrate the improved coverage. The indicators to be mapped are recorded in Table 1.

**TABLE 1:** Set targets of indicators to be analysed.

Numbers	Indicators to map	Names	Target 2023 (%)	Target 2030 (%)
1	Modern contraceptive prevalence (%)	mCPR	32.70	60.00
2	Coverage of ANC1 in the first trimester (%)	ANC1-T1	41.38	58.88
3	Coverage of ANC4 (%)	ANC4	58.40	75.00
4	Rate of delivery with a skilled birth attendant (%)	SBA	78.50	90.00

SBA, skilled birth attendant; mCPR, modern contraceptive prevalence rate; ANC, antenatal care; T1, trimester 1.

#### Spatial analysis at the health district level

The maps were analysed with a focus on achieving the 2023 target for all indicators. The approach adopted for the analysis of the maps consists of the steps below:

- Individual description at year level: the different trends according to their location in the country have been described. The percentage of HDs in each interval was calculated by emphasising the geographical location of the HDs (the areas concerned) lying below the 2023 target.
- Summary of the spatio-temporal evolution: the overall behaviour of the indicator has been described over all three years, and the rate of improvement calculated in certain cases.

#### Quantitative and temporal analysis at the scale of the health region

The charts were produced with a focus on the 2023 target for all indicators. The graphical analysis was made according to the following points:

- Description of the overall appearance of the graph.
- Calculation of the percentage of regions that have already reached the 2023 target for the indicator.
- Identification of peaks as well as depressions by specifying the regions concerned.

### Ethical considerations

This study was only concerned with routine data analysis that is anonymous and deidentified data, available in the national health management and information system (HMIS). As such, a waiver was granted by the National Ethics Committee (CNESVS) under the reference N/Ref: 265-23/MSHPCMU/CNESVS-km.

## Results

### Modern contraceptive prevalence rate

**FIGURE 1:**
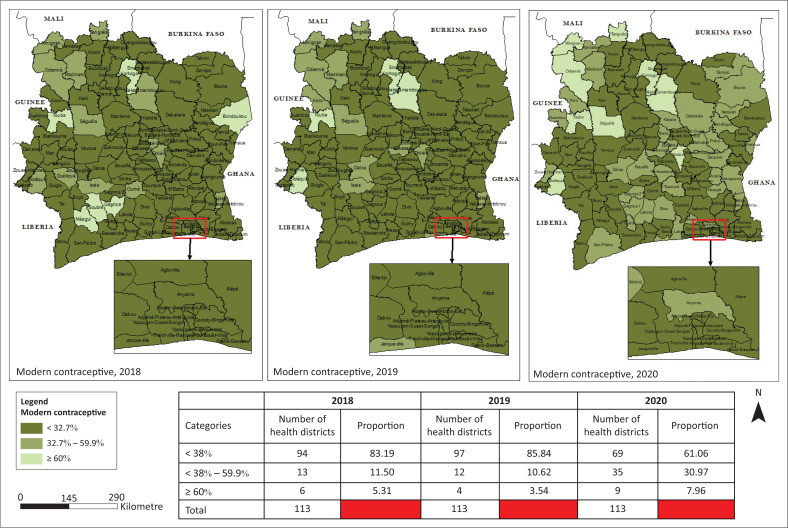
Spatio-temporal evolution of the modern contraceptive prevalence rate from 2018 to 2020.

**FIGURE 2:**
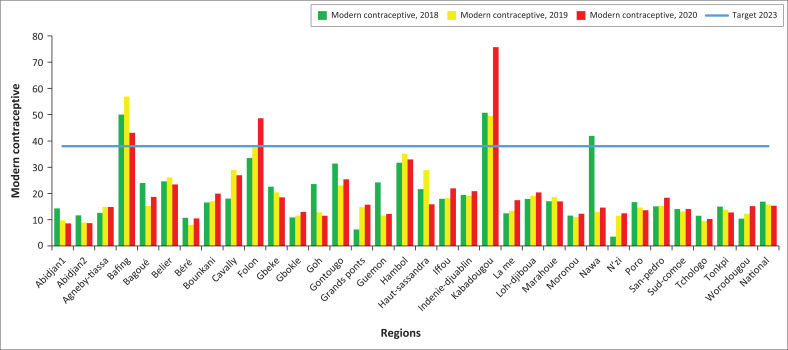
Evolution of modern contraceptive prevalence rate at regional level from 2018 to 2020.

There was a stagnant evolution of the mCPR between 2018 and 2019, then a notable evolution between 2019 and 2020 in the HDs, but most of them (61.06%) still remain below the target of 2023 (32.7%) in 2020, including all the HD of Abidjan (Figure 1). Only 3 out of 33 regions reached the 2023 target in 2020 (Figure 2). The lowest mCPR were recorded in the HD of Tiébissou, Korhogo 2, Kani, and in the health regions of Abidjan 1, Abidjan 2, Tchologo, and Béré. The probability of reaching the 2023 target by all HD seemed low.

### Proportion of ANC1 during the first trimester of pregnancy

There was a gradual evolution of the proportion of ANC1-T1 between 2018 and 2020. The proportion of HD having achieved ANC1-T1, a proportion of 41.38% (target 2023), increases from 23.01% in 2018 to 32.74% in 2020, that is, a rate of change of 42.3%. In 2020, more than 2 out of 3 of the districts in Abidjan showed an ANC1-T1 coverage of less than 41.38%. Overall, only a third of the health regions (*n* = 11/33) reached the 2023 target. However, the lowest ANC1-T1 proportion was recorded in the HDs of Korhogo 2, Transua, Kouassi Kouassikro, and the health regions of N’zi, Indenié-Djuablin and Guemon.

**FIGURE 3:**
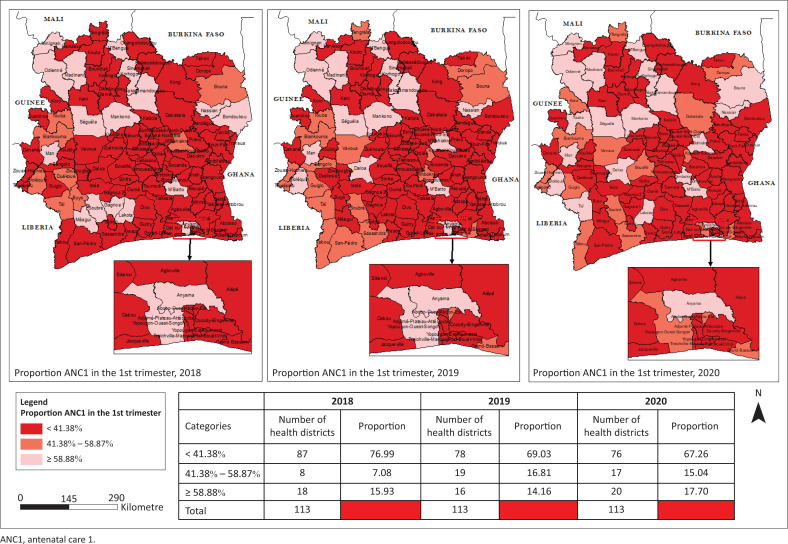
Spatio-temporal evolution of proportion of ANC1 in the first trimester in the health districts from 2018 to 2020.

**FIGURE 4:**
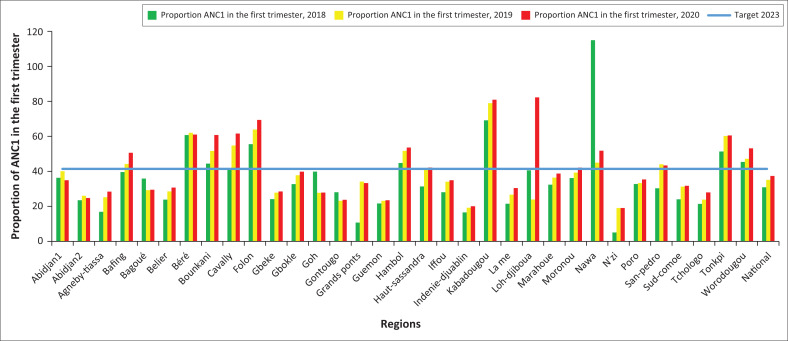
Evolution of proportion of ANC in the first trimester at regional level from 2018 to 2020.

### Coverage of ANC4

ANC4 coverage in most HDs remains below 58.4% (2023 target) but has increased significantly between 2018 and 2020. The proportion of HDs having achieved CPN4 coverage of 58.4% has increased from 13.27% in 2019 to 19.47% in 2020, that is, a growth rate of 46.72%. In 2020, only 02 districts were able to reach the target of the PNDS 2020, that is, a coverage of 90% (Target 2020, PNDS). Almost all of Abidjan’s HDs (90%) are far from the 2023 CPN4 coverage target. At the regional level, the lowest ANC4 coverage is recorded in the HDs of Korhogo 2, Kani, Yopougon-Ouest, and the health regions of Abidjan 2, Béré and Bagoué located in the south and north of Côte d’Ivoire.

**FIGURE 5:**
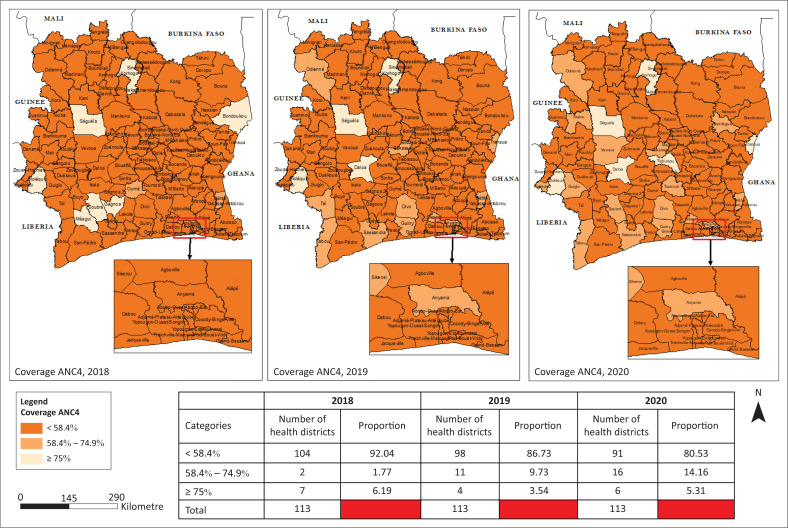
Spatio-temporal evolution of ANC4 coverage in the health districts from 2018 to 2020.

**FIGURE 6:**
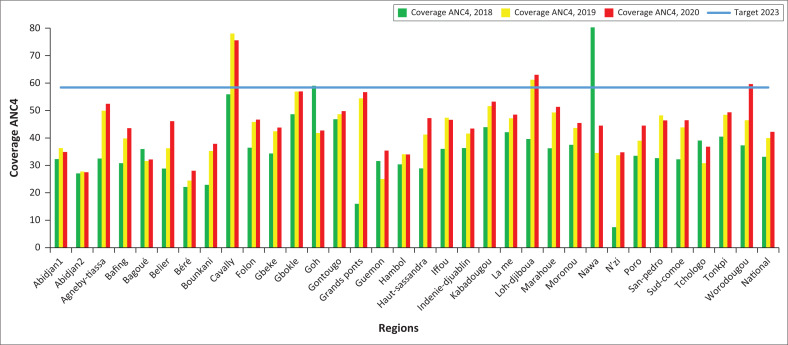
Evolution of coverage of ANC4 at regional level from 2018 to 2020.

### Rate of deliveries by a skilled birth attendant

There has been a slight improvement in the rate of births attended by qualified personnel between 2018 and 2020 at the level of the HD. In 2020, almost half of the districts (48.7%) have reached the 2023 target (78.5%). The majority, that is, 90%, of the HDs of Abidjan recorded proportions far from that target. The lowest coverage of deliveries attended by SBA was recorded in the HDs of Korhogo 2, Treichville-Marcory, Yopougon-Est, and in the health regions of Abidjan 2, Guemon and Abidjan 1. The probability of achieving the 2023 target by all HDs is high.

**FIGURE 7:**
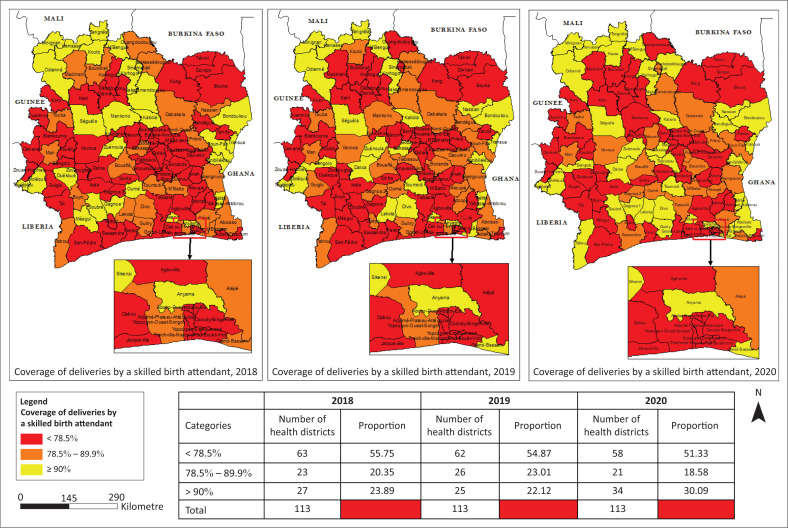
Spatio-temporal evolution of the coverage of deliveries by a skilled birth attendant from 2018 to 2020.

**FIGURE 8:**
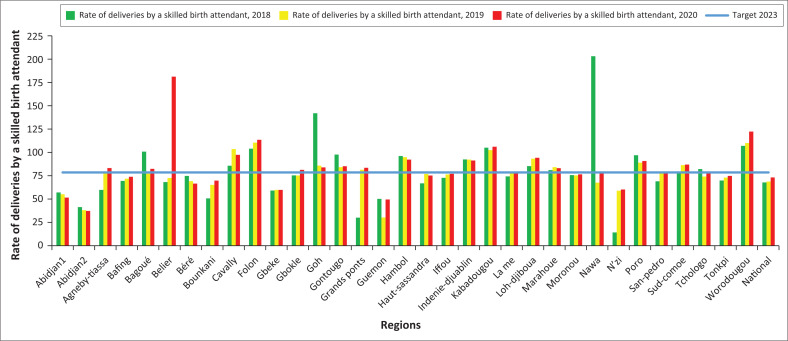
Evolution of the rate of deliveries with a skilled birth attendant at regional level from 2018 to 2020.

#### The disparities across districts and regions and the specific situation of Abidjan

There were significant disparities at the district and regional levels. In general, the lowest coverage of the indicators was recorded in the HDs of Korhogo 2 (mCPR, ANC1, ANC4 and SBA), Kani (mCPR and ANC4), and in the health regions of Béré (mCPR and ANC4), Guemon (ANC1, SBA and ANC4), and the health regions of Abidjan 1 and Abidjan 2 (mCPR, ANC4 and SBA). Finally, the five regions with the lowest levels of indicators were in the northern and southwestern regions, namely Woroba, Denguélé, Montagnes, Zanzan and Bas-Sassandra.

The distribution as well as the evolutive trends of the indicators in the regions of Abidjan showed a particular profile compared to that of the rest of the country. Indeed, most indicators were at their lowest levels in most HDs, and there was barely a variation in the level of these indicators over time.

## Discussion

This analysis of subnational progress and performance of reproductive and maternal healthcare indicators has made it possible to describe the spatial distribution of these indicators at the HD level over the past three years (from 2018 to 2020) and to analyse the temporal and spatial trends at the level of both districts and health regions.

The results showed that only one indicator, the coverage of SBA, reached the 2023 target in a significant number of HDs, while the other indicators were quite far from reaching these targets. These results were also associated with high disparities at the district and regional levels. In addition, the situation of Abidjan was specifically noticeable with very low coverage of all indicators.

The results suggested a good evolution of the SBA indicator towards the achievement of the 2023 objectives by all HDs. However, the coverage of interventions such as mCPR, ANC1 in the 1st trimester of pregnancy, and ANC4 was below the 2023 targets in several HDs. However, for ANC1 and ANC4, the positive evolutive trends over the last three years are likely to promote the achievement of the 2023 targets by all HDs. Such differences in the trends of the indicators under study are in agreement with the results of the demographic and health survey (DHS) surveys. The DHS 2021 report showed that, while the progress of the other indicators was relatively slower, the coverage of SBA increased from 45% to 59% in 2011–2012 and then to 84% in 2021, and apart from two regions (Denguélé and Woroba), the coverage at the region level varied from 75% to 97%.^3^ However, an isolated increase in SBA coverage is not sufficient for a substantial improvement in the health of the mother-child couple. Additional efforts are necessary to improve the level of the other indicators in order to strengthen the continuum of services, ranging from ANC to SBA. There were significant disparities at the district and regional levels. In general, the lowest coverage of indicators was recorded in the northern and southwestern regions, and these results are in concordance with the DHS 2021 report.^3^ Some solutions could be arrived upon for overcoming these disparities. For instance, identifying good practices implemented in HDs with a higher level of performance of mCPR, ANC1 in the 1st trimester, and ANC4 could be extended to districts where these coverages are still far from the targets. In addition, strengthening the implementation of community health interventions to increase coverage of reproductive and maternal services could also be a solution. These interventions aim to raise community awareness in order to increase demand for appropriate care and foster changes in health behaviour and practices.^12^ This implies addressing the crucial problem of the organisation and financial support of community health workers.^13^ They are a key contribution to the provision of RMNCAH care, and their integration and effective management in the health system should therefore be accelerated for optimal care at the community level. Finally, carrying out in-depth studies taking into account the socio-anthropological aspect in order to understand the reasons underlying the low levels of these indicators’ coverage could help in improving their current level.

The low progress of all indicators in Abidjan could be explained by several reasons. Firstly, maternal health services in such a capital city are largely provided by the private sector, along with tertiary-level health facilities, whose data are not adequately captured by the HMIS.^14^ Secondly, the role of the coronavirus disease 2019 (COVID-19) pandemic may also partially explain the poor performance of the indicators’ coverage in Abidjan, which was the epicentre of the epidemic and which subsequently faced a significant drop in the use of health services.^15^

All in all, these results have some programmatic implications worthy of being noted. In view of the gaps observed in the spatial distribution of the indicators under study, it is necessary to strengthen the healthcare package as a matter of priority (geographical accessibility, availability of qualified personnel, quality of care, etc.) in poorly performing HDs and regions. The health information from health facilities at the tertiary level of the health pyramid, as well as those from the private sector, should be introduced into the HMIS. This will allow an accurate epidemiological profile of the districts and health regions, especially those of large cities such as Abidjan. It is also necessary to compare the mapping of the interventions actually implemented in the HDs with the level of the indicators in order to establish a link between them and appropriately resolve any gaps that may emerge. Meanwhile, the stakeholders should continue the monitoring of the indicators in order to grasp the gaps in time and propose some corrective measures before the end of the implementation of the investment case.

We cannot fail to acknowledge the shortcoming of our study, which is mainly the use of HMIS data that are still subject to reporting errors. Although we use a validated process to improve the quality of the data, we cannot rule out other types of reporting errors.

## Conclusion

This analysis showed that the coverage of key indicators in maternal healthcare is distributed in different ways at the HD level. In fact, some HD facilities have lower coverage than others and are thus far from reaching the 2023 national targets as indicated in the Côte d’Ivoire Investment Case. All in all, two indicators had the lowest progress; the coverage of antenatal care in the first trimester and the mCPR. More efforts are needed to make sure the interventions are fully implemented, and progress monitoring should be reinforced and pursued for timely adjustments in order to reach the planned targets in 2023.
